# P-1756. Impact of order set implementation on appropriate treatment of community acquired pneumonia (CAP)

**DOI:** 10.1093/ofid/ofae631.1919

**Published:** 2025-01-29

**Authors:** Han Pham, Mitchell Stein, Lacy Worden

**Affiliations:** Ascension Borgess Hospital, Kalamazoo, Michigan; Ascension Borgess Hospital, Kalamazoo, Michigan; Corewell Health West , Grand Rapids, Michigan

## Abstract

**Background:**

The overuse and misuse of antibiotics can lead to adverse events, antibiotic resistance, and increased healthcare costs. In 2022, Ascension Borgess Hospital implemented a community acquired pneumonia (CAP) order set to help guide appropriate antimicrobial prescribing.The orderset included guideline-concordant agents and durations of therapy for CAP as well as comments empowering pharmacists to adjust total duration of therapy to account for antimicrobial doses received in the emergency department. The purpose of this study is to evaluate appropriate antimicrobial prescribing after implementing a pneumonia order set.

**Methods:**

This retrospective cohort study was conducted at a 450-bed community teaching hospital. The primary objective of this study was a composite outcome of appropriate empiric antimicrobial selection, dosing, and duration in accordance with the national guidelines. Secondary outcomes include length of hospital stay (LOS), readmission rates, mortality rates and *Clostridium difficile* infection rates. An exploratory outcome was pre-determined to evaluate pharmacists' intervention on duration of therapy, and utilization of the order-set to prescribe antibiotics. Patients were eligible for inclusion if they were 18 years or older and treated for CAP between 10/1/2021 and 8/1/2023

**Results:**

A total of 236 patients were included (118 patients per group). Significantly more patients in the post-implementation group received guideline-concordant therapy for CAP (5.9% vs 35.6%, p< 0.001). Results were heavily influenced by improvements in appropriate durations of therapy (pre- 6.8% vs post 39.9%, p< 0.001). There were no significant differences observed for LOS, 30 day readmission rates, *C. difficile* infections within 30 days, or mortality rates between groups. For exploratory endpoints, the orderset was utilized in 66.1% of patients included in the post-implementation group. Additionally, pharmacists were more likely to intervene following implementation of the orderset (22.9% v 43.2%, p < 0.001), as a result of order comments and increased awareness.

**Conclusion:**

Implementing a guideline concordant orderset significantly improved inpatient antibiotic prescribing for CAP with no difference in clinical or safety outcomes.

**Disclosures:**

**All Authors**: No reported disclosures

